# Mosquito-borne viral infections: veterinary diagnostic approach with a One Health perspective

**DOI:** 10.1128/jcm.01497-25

**Published:** 2026-03-23

**Authors:** Nicholas K. Y. Yuen

**Affiliations:** 1 Virology Laboratory, Department of Primary Industries, Elizabeth Macarthur Agricultural Institute 153388 , Menangle, New South Wales, Australia; 2 School of Veterinary Science, Faculty of Science, The University of Queensland 1974, Gatton, Queensland, Australia; Medical College of Wisconsin Pathology, Milwaukee, Wisconsin, USA

**Keywords:** vector-borne diseases, arbovirus, alphavirus, orthoflavivirus, zoonosis, diagnosis, cross-reactivity, climate change, surveillance

## Abstract

The diagnosis of mosquito-borne viral infectious diseases can be challenging, in part due to the complexity of antibody cross-reactivity between many of these viruses. This is further complicated by the unpredictable nature of climatic variability affecting disease transmission, exotic virus incursion, and the potential emergence of new strains of viruses with increased virulence. A thorough understanding of virus biology, locally relevant transmission patterns, and principles of diagnostic tests is required for the investigation of suspected clinical cases. This review provides guidance for veterinarians, researchers, and policy-makers on the diagnosis and management of alphavirus and orthoflavivirus infections in animals with a One Health perspective, including interpretation of laboratory results. Biosecurity and biosafety considerations and the zoonotic potential of mosquito-borne infections are also discussed.

## INTRODUCTION

Climatic variability has altered the transmission and exposure dynamics of vector-borne diseases (1). It is expected that the transmission of vector-borne viruses will continue to expand into previously unexposed areas, which are likely to have an increased active seasonal transmission period in endemic regions in the coming years. This is evident by the recent incursion and outbreak of Japanese encephalitis virus (JEV) in Northern and Eastern Australia in 2021–2022 (2), with the re-emergence of Murray Valley encephalitis virus (MVEV) in Victoria and Western Australia between 2022 and 2024 (3, 4), the emergence of a highly pathogenic strain of Getah virus (GETV) in China between 2017 and 2024 (5, 6), and of Western equine encephalitis virus (WEEV) in South America between 2023 and 2024 (7, 8).

While there are many different vector-borne diseases, arboviruses are of major concern, especially those transmitted by mosquitoes. Alphaviruses and orthoflaviviruses (previously known as flaviviruses [9]) are the most common mosquito-borne virus infections in animals worldwide, some of which are zoonotic (Table 1). Depending on the infectious agent, clinical signs of infected animals can range from nonspecific lethargy to arthritogenic, neurogenic, and abortogenic in nature. The consequences of arbovirus infection in production animals can be significant and widespread. It may lead to a subsequent profound impact on the economy, food supply, and biosecurity.

**TABLE 1:** List of major mosquito-borne viruses known to infect animals (primarily horses, pigs, and crocodilians)

Family	Genus	Antigenic group	Virus (10)	Clinical category (if not subclinical)	Zoonotic	Endemicity
*Togaviridae*	Alphavirus	Barmah Forest complex	Barmah Forest virus (BFV)	Arthritogenic	Yes	Australia
		Semliki Forest complex	Ross River virus (RRV)	Arthritogenic	Yes	Australia
			Getah virus (GETV)	Systemic	No	Asia
		Western equine encephalitis complex	Sindbis virus (SINV)	Arthritogenic/neurogenic	Yes	Africa, Asia, Europe, and Australasia
			Western equine encephalitis virus (WEEV)	Neurogenic	Yes	South America
		Eastern equine encephalitis complex	Eastern equine encephalitis virus (EEEV)	Neurogenic	Yes	America
		Venezuelan equine encephalitis complex	Venezuelan equine encephalitis virus (VEEV)	Neurogenic	Yes	America
*Flaviviridae*	Orthoflavivirus	Japanese encephalitis virus group	West Nile virus (WNV)	Neurogenic	Yes	Worldwide
			Murray Valley encephalitis virus (MVEV)	Neurogenic	Yes	Australia and PNG
			Japanese encephalitis virus (JEV)	Neurogenic (horse, pig) and reproductive (pig)	Yes	Asia, the Pacific, and Australasia
			Alfuy virus (ALFV)	Low-/nonpathogenic	Yes	Australia
		Kokobera virus group	Kokobera virus (KOKV)	Low-/nonpathogenic	Yes	Australia
			Stratford virus (STRV)	Low-/nonpathogenic	Yes	Australia
		Edge Hill virus group	Edge Hill virus (EHV)	Low-/nonpathogenic	Yes	Australia
		Unclassified	Fitzroy River virus (FRV)	Low-/nonpathogenic	Yes	Australia

The diagnosis of arbovirus infection, however, can be challenging. While mosquito-borne infections in animals are often subclinical, the presence of antibodies due to past exposure, vaccination, or infection may interfere with subsequent diagnosis of a clinical disease. It may also affect international travel or export/trade, which may require evidence of freedom of disease or past exposure indicated by antibodies or may require evidence of vaccination with seroconversion. This review (i) provides an update on the most common alphavirus and orthoflavivirus infections with a clinical perspective, (ii) provides guidance in the diagnosis and interpretation of laboratory results, and (iii) discusses the biosecurity concerns and One Health considerations of these mosquito-borne viruses.

## ALPHAVIRUSES

Alphavirus is the only genus member belonging to the family *Togaviridae*. Alphaviruses are enveloped spherical viruses containing a 10–12 kb nonsegmented sense single-stranded RNA genome (11). Pathogenic alphaviruses are often divided into two groups based on the primary clinical presentation: arthritogenic (often referred to as Old World alphaviruses) or neurogenic (often referred to as New World alphaviruses). However, such classification can be misleading as clinical signs vary among animal species. Of the mammalian hosts, clinical disease due to an alphavirus infection is primarily observed in horses, pigs, and humans.

### Ross River virus

RRV, which belongs to the Semliki Forest sero-complex, is the most prevalent and documented arboviral infection in horses and humans in Australia and has caused sporadic outbreaks in several Pacific nations (12–15). Transmission is typically maintained in a mosquito-marsupial endemic cycle, with horses and humans being the only confirmed susceptible dead-end hosts. While more than 40 mosquito species have been identified capable of transmitting RRV, *Culex annulirostris*, *Aedes notoscriptus*, *Ae. camptorhynchus,* and *Ae. vigilax* are the primary mosquito vectors in inland, urban, and coastal areas (16). During an outbreak, it has been suggested that RRV can be maintained in a human-mosquito-human transmission cycle, that is, without marsupial reservoir hosts (17). While not confirmed in horses, this phenomenon may be of relevance in areas densely populated with horses (e.g., training centers). Clinically affected, RRV-infected horses often present with acute febrile illnesses including lethargy, limb edema, muscle pain, and joint pain (18–21). It is important to differentiate ataxia and incoordination due to muscle stiffness/pain or joint pain as this would affect the prioritization of disease differentials. To date, there is no death reported that is directly attributable to RRV infection in horses or humans.

The apparent endemic transmission of RRV in the Pacific Islands in the absence of marsupial reservoir hosts has led to the suggestion that RRV may be an emerging infectious disease with the potential to spread worldwide (22–27). Notwithstanding the potential biosecurity concerns in countries where RRV is considered exotic, it is intriguing that clinical disease due to RRV infection has not been reported outside of Australia since the outbreak in the 1970s and 1980s. It is also worth noting that, to date, there is no published evidence of nucleic acid or antigen detection of RRV in mosquitoes in the Pacific Islands nor has there been any RRV isolates obtained. Given that only serological surveys have been conducted in the Pacific Islands, it is possible that the detection of RRV antibodies may be due to cross-reactivities to other potentially unidentified or nonpathogenic alphaviruses belonging to the Semliki Forest complex.

### Getah virus

GETV belongs to the same sero-complex as RRV, that is, the Semliki Forest complex, but is not a zoonotic agent. Based on clinical disease and viremia, it appears that transmission is maintained between mosquitoes and pigs (28), with horses and blue foxes considered dead-end hosts (29–31). To date, only genotype group III GETV has been reported to cause clinical disease. Similar to RRV, GETV has been detected in multiple mosquito species and genera, including *Culex, Aedes, Anopheles, Armigeres,* and *Ochlerotatus* spp. (32–36). Infection in horses is often mild and self-limiting; clinical disease in horses is indistinguishable from RRV infection (37). However, GETV poses a significant risk to the pig industries as infected pigs in a naïve population suffer from severe clinical diseases, with high morbidity in sows causing abortion or stillbirth and mortality in piglets with diarrhea and neurological signs (6, 38). GETV (highly pathogenic lineage III) outbreaks have recently been reported among racehorse populations in Japan (39, 40) and in piggeries and blue foxes in China (6, 31, 41). The latest GETV outbreak in piggeries in China was first reported in 2017, which continues to spread with the latest reported outbreak in Henan, Central China, in late 2024 (5, 6, 41, 42). To date, GETV has not been reported or detected outside of the Eurasian region.

### Equine encephalitis viruses

Eastern, Western, and Venezuelan equine encephalitis viruses belong to three distinct antigenic groups and are considered neurogenic alphaviruses. Clinical manifestations (e.g., ataxia and seizures) in infected horses and humans are often indistinguishable from orthoflavivirus infections. Other viral differential diagnoses include rabies virus, Australian bat lyssavirus, Hendra virus, and equine herpesvirus 1. While currently there is no approved vaccine for these viruses in humans, there are commercially available vaccines against EEEV, WEEV, and VEEV for use in horses.

Group I EEEV primarily circulates in North America and the Caribbean, while Group IIA, IIB, and III EEEV primarily circulates in Central and South America. The latest reported cases occurred in 2024 in Brazil and Ecuador (8). EEEV is maintained between *Culiseta mulanura* (mosquito vector) and avian hosts (amplifying host). The transmission or spillover of EEEV to horses and humans (dead-end hosts) occurs via a bite of an EEEV-infected bridge mosquito vector (such as *Culex* and *Aedes* genera) (43). Approximately 100–150 equine cases and 10 human cases are reported in the United States each year (44, 45). It should be noted that, as with many arboviruses, cases are likely to be underdiagnosed due to limited testing access, limited sensitivity, and clinical considerations. The case fatality rate in horses can reach approximately 90% (44) and 30%–70% in humans (46, 47).

A new WEEV lineage (lineage C) emerged in 2023–2024 causing outbreaks in South America, specifically Argentina and Uruguay (7, 8). Lineage A and B WEEV caused major outbreaks in America between the 1930s and 1990s (48, 49). However, since then, only sporadic cases have been reported in South America (7, 50) and none in the United States for >25 years (51). WEEV infections in humans are often asymptomatic or mild with a low case fatality rate (3%–14%) (47). In contrast, infected horses often suffer from neurological disease, such as seizures, with high case fatality rates (7). While *Cu. tarsalis* is the primary mosquito vector in the endemic and epidemic transmission cycle of WEEV (52), many other members in the *Culex* and *Aedes* genus are competent vectors as well (53, 54). It is worth noting that WEEV is considered a recombinant virus between EEEV and SINV due to its nucleotide and amino acid sequence similarities to EEEV in the 3′-terminal and to SINV in the E2 and E1 glycoproteins (55).

VEEV circulates throughout the Americas with regular outbreaks. The latest reported cases occurred in 2019 in Belize (8). The overall case fatality rate in humans is <1%, while in horses, it can reach up to 70% (56). The endemic transmission cycle of VEEV (subtype ID and IE) is maintained between sylvatic rodents and *Culex* spp. mosquitoes (57). In contrast to EEEV and WEEV, horses serve as amplifying hosts in the epidemic transmission cycle of VEEV (subtypes IAB and IC), which can lead to spillover events to humans (dead-end host) through the bite of an infected mosquito (e.g., *Culex* spp., *Aedes* spp., and *Psorophora* spp.) (57). Of note, VEEV is listed as a select agent in the United States (58).

### Sindbis virus

SINV belongs to the Western equine encephalitis complex and is found across Africa, Asia, Europe, and Australasia. Transmission of SINV is maintained between *Culex* spp. mosquitoes (primarily *Cu. torrentium*) and birds, with horses and humans being the dead-end spillover hosts after being bitten by an infected bridge vector, such as *Aedes* spp. mosquitoes (59, 60). While there are five SINV genotypes, only infection with SINV-1, which circulates in northern Europe and Africa, has been reported to be associated with disease in horses and humans (61–63). Disease in humans is often referred to as Ockelbo disease in Sweden and Pogosta disease in Finland (61). Historically, clinical disease associated with SINV infection has been classified as arthritogenic, similar to that of RRV infection. However, fatal neurological disease due to SINV infection has recently been reported in both horses and humans in South Africa (64, 65).

## ORTHOFLAVIVIRUSES

Orthoflavivirus belongs to the family *Flaviviridae*. Orthoflaviviruses are enveloped spherical viruses (diameter of ~50 nm) containing a 9.2–11 kb single positive-stranded RNA genome (9, 66). The primary clinical presentations due to infection of orthoflaviviruses in susceptible hosts are neurological disease and reproductive failure, often indistinguishable from diseases caused by neurotropic alphaviruses. Of the mammalian and reptilian hosts, clinical disease due to orthoflavivirus infection is primarily observed in horses, pigs, crocodilians, and humans.

### West Nile virus

WNV is classified into 9 lineages, with only lineages 1, 2, and 7 associated with disease and outbreaks in humans and animals (67–69). WNV lineage 1 can be divided into 3 clades. Clade 1a is widespread, circulating in many countries, except Australasia; clade 1b, also known as the Kunjin virus, circulates in Australia; clade 1c is found in India (69, 70). WNV lineage 2 circulates in Europe, Madagascar, and Africa (69, 70). The endemic transmission of WNV is maintained between *Culex* spp. mosquitoes and avian hosts (e.g., waterbirds, sparrows, and robins). Crocodilians are competent amplifying hosts for WNV (71, 72), while horses and humans are considered dead-end hosts (73). While there is serological evidence of WNV infection in pigs (74–76), upon experimental infection, pigs are not susceptible to disease and are considered dead-end hosts (77, 78).

Virulence or disease severity is strain-dependent. Infection with WNV (lineage I, clade 1a; lineage II) in humans and horses can cause severe fatal neurological disease (79–81), whereas the endemic Australian WNV_KUN_ strain (lineage I, clade 1b) only causes mild disease in infected humans and horses (82–84). Overall, the case fatality rate in humans is approximately 10% (85). In horses, the overall case fatality rate is approximately 40% (86). It is worth noting that the case fatality rate for WNV_KUN-NSW2011_ (lineage I, clade 1b) in horses was 9% (87). Approximately 200 equine cases and 2,000 human cases are reported in the United States each year (44, 88), with epidemics occurring approximately every 3–5 years (89). Vaccines are commercially available for use in horses in some countries.

WNV infection in American alligator (*Alligator mississippiensis*) may manifest in three forms: cutaneous (e.g., “pix” skin lesion), gastrointestinal (e.g., anorexia and bloating), or neurological (e.g., ataxia, circling, and unbalanced swimming) (90–92), while only cutaneous lesions have been reported in infected saltwater crocodile (*Crocodylus porosus*) (93, 94). Vector-free WNV transmission via excreta (i.e., fecal-oral route) among crocodilians has been demonstrated (71, 72, 95).

### Japanese encephalitis virus

JEV is widespread in many parts of Asia, the Pacific, and Australasia. While there is only one serotype of JEV, based on the phylogenetic analysis of the envelope (E) gene, five genotypes exist. While the annual incidence of JEV infection in animals is not known, it has been estimated that globally there are 60,000–100,000 human cases each year (96, 97). In 2022, an incursion of JEV in mainland Australia caused widespread disease in piggeries and humans (2). However, to date, there is no published report of confirmed diagnosis of JEV infection in horses in mainland Australia. Since the incursion, there was a low-moderate level (15%) of JEV sero-prevalence in horses in Queensland (73). The endemic transmission of JEV is maintained between amplifying hosts (waterbirds and pigs) and *Culex* spp. mosquitoes (primarily *Cu. tritaeniorhynchus* in Asia and *Cu. annulirostris* in Australia) (98–101). In the epidemic transmission cycle, humans and horses are considered dead-end hosts. Virus shedding via oral fluid in pigs has been demonstrated as well (102). While seroconversions have occasionally been reported in other animal species (such as sheep, cattle, and alpacas), clinical disease is rarely observed, and these species are considered to be incidental dead-end hosts (103, 104).

Upon infection, pigs, horses, and humans are susceptible to disease. Clinical manifestations in horses and humans are characterized by neurological disease, such as ataxia and seizures. Case fatality rates in humans and horses are approximately 20%–30% (96) and 5%–15% (105), respectively. Infection in adult pigs is subclinical. Naïve pregnant sows and gilts may abort or deliver stillborn or mummified fetuses. Piglets born alive from infected pregnant sows/gilts often suffer from muscle fasciculations, tremors, and seizures, with case fatality rates as high as 100%. An abortion storm is possible in naïve populations under intensive farming conditions. It has been reported that abortion caused by JEV in pigs does not appear to affect fertility or reproductive performance in future pregnancies (106). Infected boar may experience orchitis, leading to temporary reduced fertility (107, 108). Differential diagnoses in pigs include GETV, porcine circovirus, porcine respiratory and reproductive syndrome virus, atypical porcine pestiviruses, classical swine fever virus, and African swine fever virus. Several commercially available vaccines are available to protect horses, pigs, and humans against JEV.

### Murray Valley encephalitis virus

MVEV is a member of the Japanese encephalitis sero-complex and is endemic in Australia and Papua New Guinea. Transmission of MVEV is maintained between waterbirds and *Culex* spp. mosquitoes (98). Horses and humans are susceptible dead-end hosts in the epidemic transmission cycle. Serological surveys in horses performed in 2013 and 2023, after the WNV and JEV outbreaks, respectively, demonstrated low seroprevalence of MVEV in horses in eastern Australia (73, 109). Since the largest MVEV outbreak recorded in 1974 with an approximately 20% case fatality rate (110), MVEV continues to cause sporadic fatal neurological illnesses in horses (3, 111, 112) and humans (3, 4). MVEV has recently re-emerged in Australia, causing outbreaks in horses and humans in 2011 and in humans in 2023 (3, 113–115).

## LOW-/NONPATHOGENEIC MOSQUITO-BORNE VIRUSES OF IMPORTANCE

From a diagnostic perspective, other co-circulating low-/nonpathogenic viruses belonging to the same serogroup or antigenic complex should be considered as well primarily due to antibody cross-reactivities affecting the interpretation of serological test results. Often, these alphaviruses and orthoflaviviruses are considered low- or nonpathogenic as infection of these viruses is evident by seroconversion but rarely causes clinical disease in animals. Knowledge of locally relevant epidemiology of these viruses is required. Examples are provided below.

Saint Louis encephalitis virus (SLEV), dengue virus (DENV), and Zika virus (ZIKV) are orthoflaviviruses of medical importance globally. Seroconversions have been detected in horses without clinical disease (116, 117). However, a rare case of neurological disease in an SLEV-infected horse was reported in Brazil (118). Of importance, SLEV and WNV belong to the same serogroup (JE antigenic complex) which co-circulates in the Americas. Serological cross-reactivities are also observed between DENV and ZIKV, which circulates worldwide.

Many low- or nonpathogenic orthoflaviviruses co-circulate in Australia, such as Alfuy virus (ALFV), Stradford virus (STRV), Kokobera virus (KOKV), and Fitzroy River virus (FRV). Only KOKV and FRV have caused clinical disease in humans (119, 120). However, seroconversion to these viruses in horses has been reported (109, 120). Of importance, antibody cross-reactivities are observed between ALFV, JEV, WNV, and MVEV (JE antigenic complex).

## DIAGNOSIS AND LABORATORY RESULT INTERPRETATION

**Fig 1:**
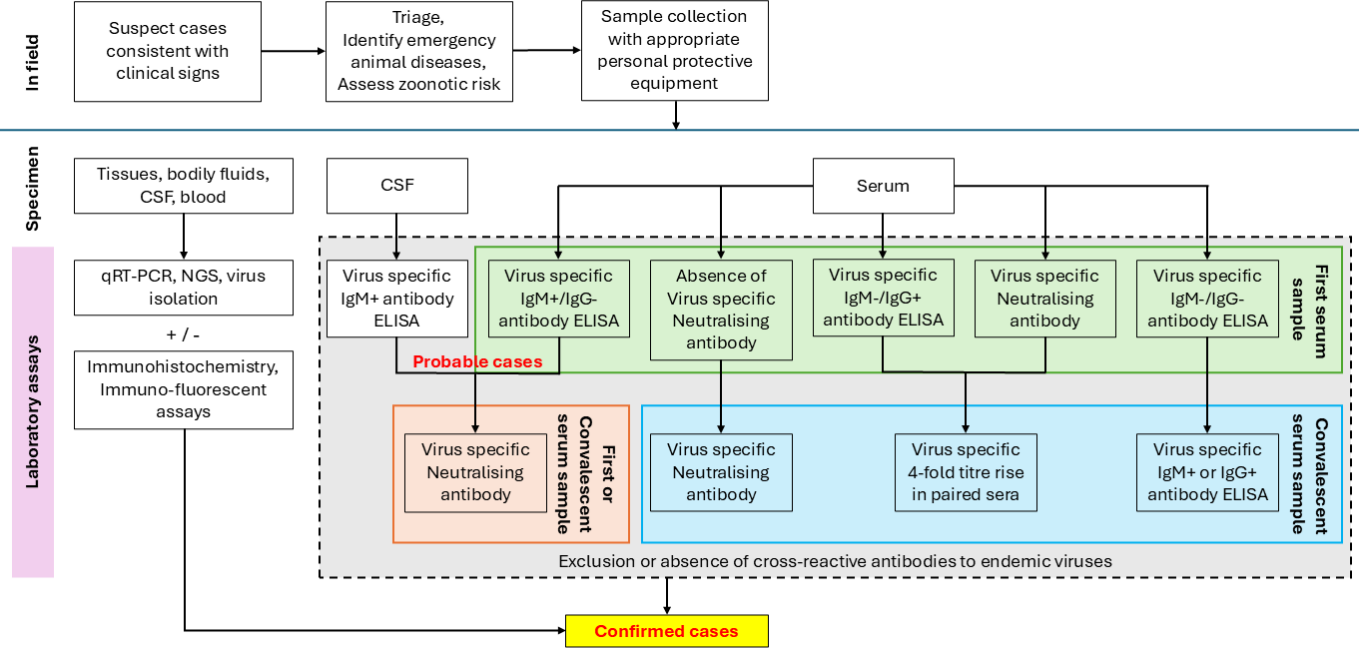
Diagnostic workflow for suspected alphavirus or orthoflavivirus infection. First (acute) blood sample should be collected within 24–48 h of clinical onset. A convalescent blood sample should be collected 10–14 days after initial sampling. For a serology-based assay, the presence of cross-reactive antibodies to endemic viruses must be excluded or resolved for each sample, by means of neutralizing antibody titer. The same serological assay must be used to compare titer fold changes in paired serum samples. Immuno-labeling assays may be used as an adjunct for confirmation of direct detection.

The definitive diagnosis of alphavirus or orthoflavivirus infection requires either confirmation of seroconversion, virus isolation, or molecular detection of viral nucleic acid in the sample corresponding with the time of clinical disease (105, 121–123) (Fig. 1).

In a live horse, the detection of viral nucleic acid by qRT-PCR (molecular testing) or isolation of the causal virus in blood/plasma, cerebrospinal fluid (CSF, in the case of neurological disease), and synovial fluid (in the case of arthritogenic disease) would provide definitive diagnosis of infection (Table 2). Next-generation sequencing would also provide definitive diagnosis and is often used in virus discovery and characterization, especially for genotyping in an outbreak situation. However, it may be cost-prohibitive and time-consuming for use as a primary diagnostic modality. Viremia of pathogenic alphaviruses and orthoflaviviruses in horses is short-lived (between 48 and 72 h post-infection) and often undetectable by the time of clinical onset (21, 84, 124). Therefore, a negative result does not definitively rule out recent infection. For the same reason, virus isolation and virus antigen capture ELISA testing are often unrewarding.

**TABLE 2:** Summary of laboratory methodologies for the diagnosis of mosquito-borne infection in animals^*c*^

Type of test	Test	Required sample type	Pros	Cons
Serology	Virus neutralization test (VNT): (1) Micro-neutralization test or (2) Plaque reduction neutralization test (PRNT)	Serum	Allows definitive diagnosis/confirmation of infection	Requires a convalescent blood sample Assay turnaround time ~3–7 days Requires use of cell lines and infectious virus Result interpretations can be complex due to potential antibody cross-reactivity Requires BCL3 facilities to perform assays with infectious BCL3 agents
	Antibody-based ELISAs (e.g., indirect and competitive)	Serum, plasma, and CSF^*a*^	Turnaround time of 1–1.5 days Typing of antibodies Useful as a screening test	Do not provide definitive diagnosis Affected by antibody cross-reactivity Require VNT/PRNT confirmatory test
Antigen detection	Virus isolation	Serum, plasma, and tissues^*b*^	Allows definitive diagnosis/confirmation of infection if culture-positive	Requires use of appropriate cell lines Can take several weeks before the result is available Negative result does not rule out infection Short-viremic phase in horses
	Antigen capture ELISA	Serum, plasma, and tissues^*b*^	Allows definitive diagnosis/confirmation of infection if the antigen is detected Turnaround time of 1 day	Negative result does not rule out infection Short-viremic phase in horses
	Immunohistochemistry and immunofluorescent assays	Tissues^*b*^	Allow definitive diagnosis/confirmation of infection if the antigen is detected	Sample collection from deceased animals Negative result does not rule out infection
Molecular	qRT-PCR and next-generation sequencing	Whole blood, plasma, and tissues^*b*^	Allow definitive diagnosis/confirmation of infection if nucleic acid is detected Provide semi-quantitative results Turnaround time of 0.5 day	Negative result does not rule out infection Short-viremic phase in horses

^*a*^ Suitable for neuropathogenic alphaviruses and orthoflaviviruses.

^*b*^ Tissues/bodily fluid should be submitted chilled (not frozen). Histological assessment (including immuno-labeling assays) may help with the selection of an appropriate sample type. Joint fluid may be suitable for the detection of arthritogenic alphaviruses. Brain, CSF, spleen, thoracic fluid, liver, placenta, and any fetal tissue/fluid may be suitable for the detection of abortogenic and neuropathogenic alphaviruses and orthoflaviviruses.

^*c*^ BCL = Biocontainment level; CSF = cerebrospinal fluid.

In contrast, given the role of pigs as amplifiers in the epidemiology of GETV and JEV, infectious virus and nucleic acid are often detected by virus isolation and molecular methods, respectively, in samples from affected litters/piglets. Depending on the stage of viremia, the virus may be detectable in the blood of affected adult pigs as well. Infectious virus may persist in the tonsil of pigs for >21 days and be detectable in the oral fluid (102).

In case of post-mortem assessment in animals, the appropriate sample types are brain, CSF, synovial fluid, spleen, thoracic fluid, liver, placenta, and any fetal tissue/fluid (125). All samples should be submitted chilled, not frozen, to preserve cellular structural integrity for histological assessment and sensitivity of some laboratory assays (e.g., virus isolation). Representative tissue samples should also be preserved in 10% buffered formalin for 48–72 h for histological assessment (including immuno-labeling assays).

Given the low likelihood of direct detection via molecular approaches and virus isolation in horses, serology-based (indirect detection) diagnostic tests are often preferred (Table 2; Fig. 1). Paired serum samples are required for serological testing by ELISA or VNT (73, 109, 126, 127). For this purpose, convalescent blood samples collected at least 7–10 days (ideally after at least 14 days) after initial sampling must be available to demonstrate a significant increase in antibody titer due to recent infection. The initial blood sample should be collected at the acute phase of disease, that is, at the time of onset of clinical signs. If antibody is present in the initial serum sample, a four-fold or greater increase in antibody titer is required to demonstrate recent infection. When antibody is not detected in the first serum sample, detection of antibody in the convalescent sample is significant.

However, there can be substantial cross-reactivity and reduced specificity, especially if there are antibodies against more than one virus belonging to the same antigenic group or sero-complex present in the serum, for example, between RRV and GETV (Semliki Forest virus sero-complex) and between WNV, JEV, and MVEV (JE sero-complex) (73, 128, 129). In theory, assays for low-/nonpathogenic alphaviruses and orthoflaviviruses should also be considered, although there are no ELISAs routinely available for these viruses. Result interpretations for these tests can be complex due to the potential for infection with endemic viruses belonging to the same serogroup. However, a four-fold or greater difference in neutralizing antibody titer (determined by VNT) between two viruses is usually interpreted as indicating that the current/recent infection is likely to be due to the virus with a higher antibody titer. Co-infection and/or past exposure to other closely related alphaviruses or orthoflaviviruses should be considered as well. In addition, vaccination history should also be considered. Maternal antibodies may be present in the serum/plasma of newborn or young animals (< 6 months of age) due to the transfer of passive immunity via colostrum from seropositive dams (73, 127). Due to the complexity of test selection, interpretation of results, and the required taxological knowledge of circulating endemic viruses (both pathogenic and nonpathogenic), veterinarians are encouraged to seek expert advice when investigating potential alphavirus or orthoflavivirus disease.

It should be noted that VNT (often micro-neutralization test or plaque reduction neutralization test) requires use of cell lines and infectious viruses. The assay usually requires at least 3–7 days of incubation before the antibody titer can be determined. Taking into account the requirement of a convalescent sample means that a definitive diagnosis may not be available until at least 21 days after clinical presentation.

The current major bottleneck is that there are limited diagnostic assays available for the detection of antibodies against many of the cross-reactive orthoflaviviruses and that VNT on these viruses is infrequently conducted. Some arboviruses, such as JEV and EEVs, are classified as biocontainment level 3 organisms in some countries, which further limits the availability of testing by VNT. However, this may be overcome by using a chimeric virus, for example BinJ-JEV, which is a biocontainment level 2 organism that has been proven to only infect invertebrate (mosquito) cells, making it safe to handle while offering comparable neutralizing antibody titer results to that of VNT using wild-type JEV (73, 130).

One benefit of ELISA is the ability to identify immunoglobulin M (IgM) antibodies in the serum or CSF, which would indicate recent infection (131). However, it should be noted that, while not confirmed for alphaviruses, serum IgM in horses can persist for up to at least 3 months post-infection or vaccination (132–134). The demonstration of virus-specific IgM, without IgG, in the serum and/or CSF is suggestive of relatively recent infection, though it may not be definitive due to potential antibody cross-reactivities and/or persistence (132–134). The presence of IgM in CSF or serum with suggestive clinical signs may be interpreted as probable cases.

## BIOSECURITY AND BIOSAFETY

The transmission dynamics of alphaviruses and orthoflaviviruses, as mosquito-borne viruses, are affected by the biology, movement, and interaction between mosquitoes, reservoir hosts, and susceptible hosts. As a result of climate change, the temperatures in the temperate regions are increasing with increased extreme weather events, which favor the spread and survival of mosquitoes and the migration of reservoir hosts (1). Overall, this is likely to (i) increase the risk of spillover and outbreak of endemic mosquito-borne diseases to susceptible hosts, such as horses, (ii) extend the active transmission season of mosquito-borne diseases, (iii) increase the likelihood of an incursion and establishment of exotic mosquito species, such as *Ae. albopictus* and *Ae. aegypti*, that are efficient vectors for mosquito-borne viruses, and (iv) allow viruses to overwinter in mosquito eggs and larvae and then proliferate in spring/summer (131, 135).

Using Australasia as an example, endemic circulation of JEV had only been confirmed in the Torres Straits and Cape York Peninsula (northern Australia), but incursion into mainland Australia was not established until 2022 (2, 136). This demonstrates the importance of vigilance toward mosquito-borne diseases that have not yet established in Australia but are sporadically detected in the tropical region of Australia. Importantly, despite not yet becoming established in mainland Australia, the mosquito vector *Ae. albopictus* is endemic in the Torres Straits (137) and has been intercepted multiple times in international ports throughout Australia (138). With climate change and the increased tolerance to temperate climate of *Ae. albopictus*, the likelihood of an incursion and establishment of this mosquito species (and *Ae. aegypti*), and the associated disease risks, should not be underestimated (135).

While vaccines have been approved for use in livestock in some countries, they are only commercially available for some alphaviruses (EEEV, WEEV, and VEEV) and orthoflaviviruses (WNV and JEV). Currently, in Australasia, there are no approved vaccines to prevent, or therapeutics to treat, mosquito-borne virus infections in animals. Therefore, a proactive preventative measure is required to minimize infection of mosquito-borne diseases. Examples include the use of insect repellents; stabling horses during dusk and dawn, when mosquito activities peak; and minimizing mosquito breeding grounds by removing water pools.

It should be noted that vaccination would affect the interpretation of serological test results unless a differentiating infected and vaccinated animal (DIVA) assay is available. A DIVA assay detects antibodies that are present in naturally infected animals but not in vaccinated animals. To achieve this, the vaccine must lack at least one immunogenic component of the virus. Using the recombinant canarypox vectored WNV vaccine as an example, the virus only displays the prM and E proteins of WNV (139). Therefore, when used as a vaccine, the nonstructural proteins of WNV (e.g., nsP1) may be used as antigens for DIVA assays. It is expected that vaccinated animals (assuming without any prior natural exposure to orthoflaviviruses) will only generate antibodies against prM and E proteins, but not other structural or nonstructural proteins of WNV. However, in reality, the diagnostic challenge of antibody cross-reactivities to other closely related orthoflaviviruses remains.

While most of the viruses discussed here are transferred by the bite of an infected mosquito, some carry significant zoonotic risk. For example, infectious WNV and JEV are excreted via cloaca in fecal matter in crocodilians and via tonsils in oral fluid of pigs, respectively, which may be responsible for vector-free horizontal transmission during an outbreak under intensive farming conditions (71, 72, 95, 102, 140). There are also reports of laboratory-acquired WNV infection (141). Therefore, farm staff, laboratory workers, and owners should be educated to identify early clinical signs related to mosquito-borne infections in animals.

Horses may be used as sentinels to monitor the transmission of mosquito-borne viruses. This can be achieved by several surveillance monitoring systems. In the United States, the transmission of EEEV is monitored through passive targeted surveillance, whereby detection of EEEV is nationally notifiable (44, 45). This detection method relies on reporting of laboratory diagnosis of clinical cases. While it is cost-effective, mild diseases may be under-reported. There is also mounting evidence that active nontargeted surveillance via longitudinal sampling of strategically located sentinel horse populations will likely provide locally relevant real-time virus transmission patterns (127, 142). This will also allow early detection of the potential incursion of exotic viruses.

## GAPS AND CONCLUSION

This review represents a comprehensive review of common alphaviruses and orthoflaviviruses affecting animals, many of which are also human pathogens. It provides guidance on the diagnosis of disease caused by alphaviruses and orthoflaviviruses and insights into the potential complications for current diagnostics. The interpretation of diagnostic test results is multifaceted and requires knowledge of virus biology and the principles of relevant laboratory assays. Given the frequency of subclinical infections with alphaviruses and orthoflaviviruses, the clinical significance of diagnostic test results should always be interpreted in light of clinical history on a case-by-case basis.

Interpretation of diagnostic tests is complex, especially due to serological cross-reactivities, making definitive diagnosis of mosquito-borne infection very challenging and time-consuming at times. Future assay development and validation of orthoflavivirus serological tests will improve current veterinary diagnostic capabilities. Development of a DIVA assay will allow differentiation of antibodies produced by vaccination or infection. This would be important in the context of export certification where demonstration of a “freedom of disease” status is required.

The transmission of mosquito-borne viruses is dynamic and varies across the globe due to its vast landscapes and differing weather patterns. The use of horses as sentinel animals suggested previously (73, 127, 131) may inform veterinarians, infectious diseases physicians, and public health officials with an up-to-date locally relevant transmission pattern of mosquito-borne diseases to allow informed veterinary and public health policy-related decisions to be made.

Climate change has contributed to the unpredictable nature of vector-borne diseases. The emergence of new strains of endemic viruses with increased virulence or incursion of exotic viruses is a continuous threat. Veterinarians should be vigilant and consider the possibility of the zoonotic potential of differential diagnoses to protect themselves, workers, and animal owners from immediate health concerns. If required, veterinarians should contact the emergency animal disease authorities, follow the directive/guidance given by government officials, and collect appropriate samples (with personal protective equipment) to exclude emerging animal diseases and/or endemic diseases of high consequences to human/animal health.
